# Expression differences of programmed death ligand 1 in de-novo and recurrent glioblastoma multiforme

**DOI:** 10.18632/oncotarget.18819

**Published:** 2017-06-28

**Authors:** Sabrina Heynckes, Annette Gaebelein, Gerrit Haaker, Jürgen Grauvogel, Pamela Franco, Irina Mader, Maria Stella Carro, Marco Prinz, Daniel Delev, Oliver Schnell, Dieter Henrik Heiland

**Affiliations:** ^1^ Department of Neurosurgery, Medical Center, University of Freiburg, Freiburg, Germany; ^2^ Department of Neuroradiology, Medical Center, University of Freiburg, Freiburg, Germany; ^3^ BIOSS Centre for Biological Signaling Studies, University of Freiburg, Freiburg, Germany; ^4^ Institute of Neuropathology, Medical Center, University of Freiburg, Freiburg, Germany; ^5^ Faculty of Medicine, University of Freiburg, Freiburg, Germany

**Keywords:** immune checkpoints, PD-L1, GBM, recurrent GBM

## Abstract

The biology of recurrent glioblastoma multiforme (GBM) is a dynamic process influenced by selection pressure induced by different antitumoural therapies. The poor clinical outcome of tumours in the recurrent stage necessitates the development of effective therapeutic strategies. Checkpoint-inhibition (PD1/PD-L1 Inhibition) is a hallmark of immunotherapy being investigated in ongoing clinical trials. The purpose of this study was to analyse the *PD-L1* expression in de-novo and recurrent glioblastoma multiforme and to explore associated genetic alterations and clinical traits. We show that *PD-L1* expression was reduced in recurrent GBM in comparison to de-novo GBM. Additionally, patients who received an extended dose of temozolomide (TMZ) chemotherapy showed a significantly reduced level of *PD-L1* expression in the recurrence stage compared to the corresponding de-novo tumour. Our findings may provide an explanation for potentially lower response to immunotherapy in the recurrent stage due to the reduced expression of the therapeutic target *PD-L1*.

## INTRODUCTION

Glioblastoma multiforme (GBM) is the most common type of malignant brain tumour, which is characterised by poor clinical outcome and short survival time, rarely longer than 14 months [1]. During the last decades extensive efforts have been made to develop new treatment strategies without significantly improving the poor clinical course of GBM patients [2, 3]. Until now, the “gold standard” in glioblastoma multiforme treatment remains surgery plus adjuvant combined chemoradiotherapy introduced by Stupp et al. in 2005 [4]. Especially for the treatment of recurrent GBM, effective therapeutic options are limited and have not yet been well examined.

Recently, a novel class of immunotherapies - the immune checkpoint inhibitors - have successfully influenced the treatment of a large variety of different solid cancer types [5]. These inhibitors (CTLA-4, PD-L1, PD-1) are able to block the immune checkpoint signalling, which leads to a T-cell response against the tumour [6–8]. GBM contain frequent genetic and epigenetic alterations and initiate numerous of potential neoantigens [9]. These neoantigens are recognized by the immune system followed by a T-cell based antitumoural immune response [10]. From this standpoint, new treatment strategies are being currently investigated and tested in clinical trials. Those treatments mainly target and inhibit the programmed death-ligand 1 (*PD-L1*) on the tumour cell surface or the programmed death-ligand protein 1 (*PD-1*) receptor on the T-Cell in attempt to support antitumoural immune response.

Recurrent GBM show an increased number of genetic alterations, which are potentially induced by radio- and chemotherapy [11] and may result in a stronger vulnerability towards recognition and attack by the immune system [12, 13]. The occurrence of PD-L1 expression in GBM patients has been shown in several recent studies, which mainly focused on newly diagnosed GBM patients [6, 14, 15]. However, little is known about the PD-L1 expression in recurrent glioblastoma. Berghoff et al., 2014 analysed by immunohistochemistry a small subcohort of 18 patients and reported a lower frequency of PD-L1 expression in recurrent GBM compared to newly diagnosed GBM [15]. A recent study analysed the PD-1 and PD-L1 protein-level in 16 recurrent mixed (primary and secondary) GBM patients by immunostaining. A non-significant increase of PD-L1 protein-level in recurrent specimens was reported [16].

The purpose of this study was to analyse PD-L1 expression on mRNA and protein-level in patients with de-novo and recurrent glioblastoma multiforme. First, we investigated the PD-L1 expression in GBM at different stages (64 cases) (de-novo tumour (n=64), first (n=38), second (n=18) and third recurrence (n=10)). Secondly, de-novo and recurrent samples from the same patients (38 cases) were analysed and the change of PD-L1 expression over time within the same tumour was examined. Finally, the laboratory results were correlated with clinical data and confirmed by taking the results of 874 patients of 6 independent cohorts from different publicly available databases into consideration.

## RESULTS

**Figure 1:**
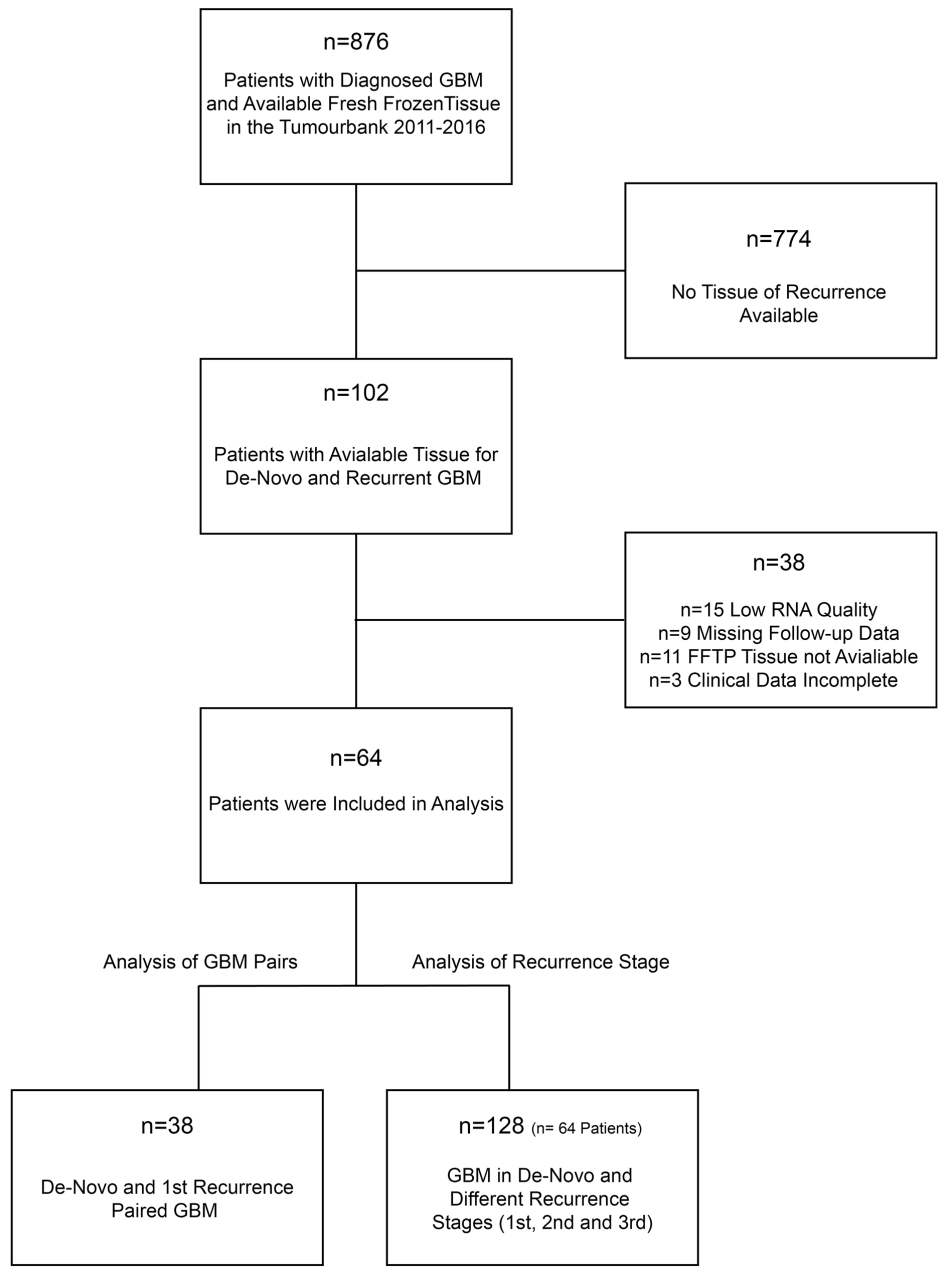
Workflow for study screening and patients recruitment

Tissue samples of 876 patients were screened for paired samples of de-novo and recurrent tumours, which were acquired during surgical resection between 2011-2016. 102 patients with matched samples (de-novo and recurrent specimens) were identified. Of these, 64 patients achieved all quality criteria and were included in the study (Figure 1). The mean age of the cohort was 48.66 (±18.33) years. All patients underwent at least one recurrent surgery at the Department of Neurosurgery, Medical-Center, University of Freiburg. 38 patients had available tissue from the first recurrent surgery, 21 (32.81%) patients received a surgery of the 2^nd^ recurrence and 5 (7.81%) patients received a surgery of the 3^rd^ recurrence.

### Analysis of mRNA expression and protein-level in recurrent GBM

**Figure 2:**
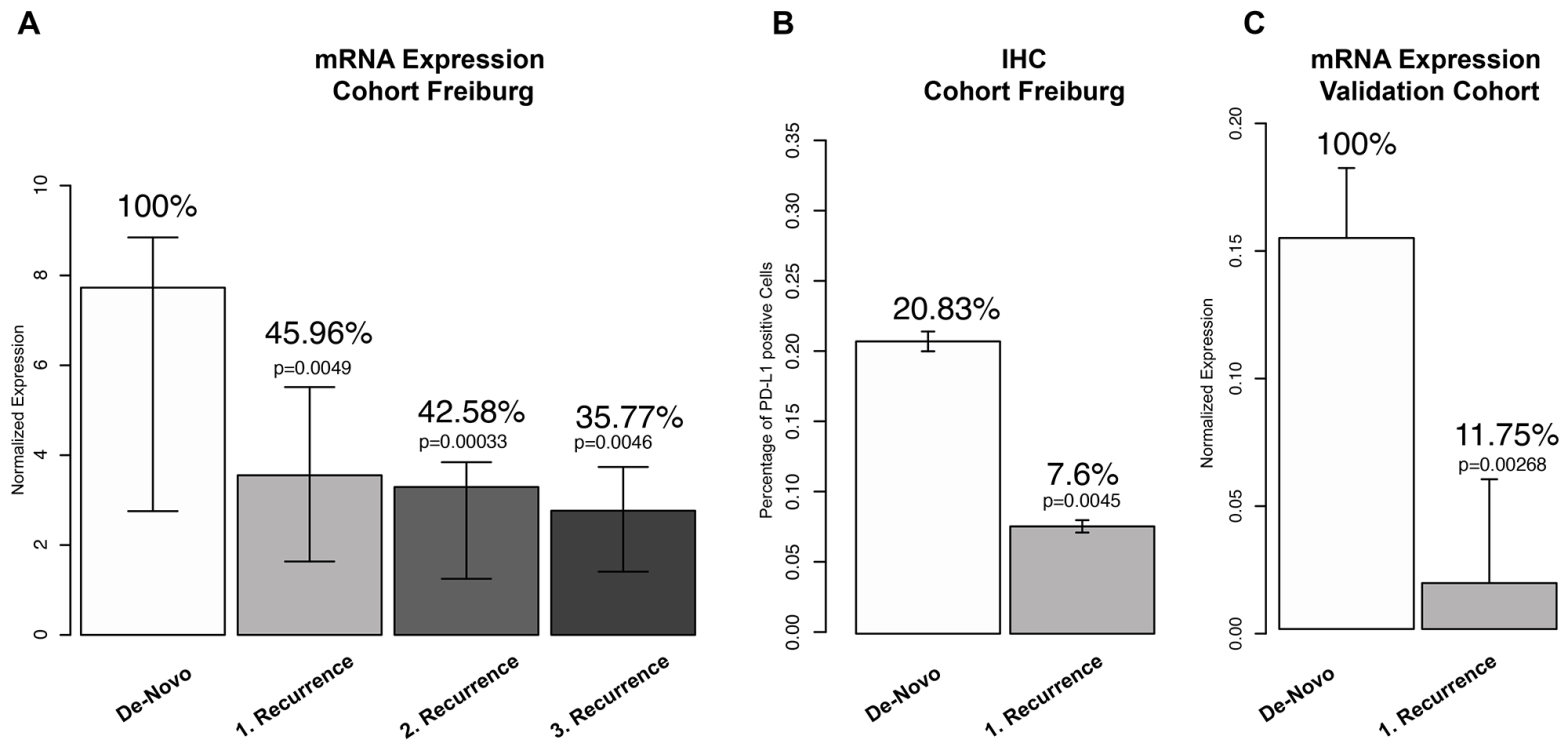
**(A)** Analysis of PD-L1 mRNA expression in de-novo GBM and different recurrence stages. **(B)** Immunohistochemistry (IHC) of PD-L1 in paired de-novo/1^st^ recurrent GBM. **(C)** PD-L1 expression values of 874 patients from public available databases in de-novo and recurrent GBM.

*PD-L1* expression of 128 tissue samples (64 GBM patients) was analysed by qRT-PCR. De-novo tumours from all patients (n=64) showed highly variable expression values (median 7.52 normalized expression (NE) IQR 2.72-8.89 NE). Samples of the first recurrence (n=38, median expression 3,45 NE IQR 1.89-5.76 NE) showed a decreased median expression of PD-L1 of 54.04% in comparison to de-novo GBM (Figure 2A). This difference was statistically significant (p=0.0041). In the 2^nd^ recurrence PD-L1 expression (3.20 NE IQR 1.3-3.9) was further reduced (3.38 %) in comparison to the fist recurrence (53% less expression of PD-L1 in comparison to de-novo tumours, p=0.00033). Tumours of the 3^rd^ recurrence (2.63 NE IQR 1.35-3.57) showed a significant difference in their PD-L1 expression (64.3% less expression of PD-L1 in comparison to de-novo tumours, p=0.0046). Additionally, immunohistochemistry (IHC) of PD-L1 was performed and showed a reduction of 66.71% (p=0.0045) in the first recurrence, Figure 2B. In a second step, we aimed to validate our findings based on publicity available data. We performed gene expression analysis of *PD-L1* of 6 different public-available datasets, Figure 1C. A total number of 874 GBM (731 de-novo and 143 recurrent glioma) were analysed and showed a significant down-regulation of PD-L1 in recurrent glioblastoma (reduction of 88,25% p=0.00268), Figure 1C. Summarized, the results reported a significant down-regulation of the checkpoint molecule PD-L1 in recurrent glioblastoma.

### Immunohistochemistry (IHC) and immunofluorescence (IF) of recurrent GBM

**Figure 3:**
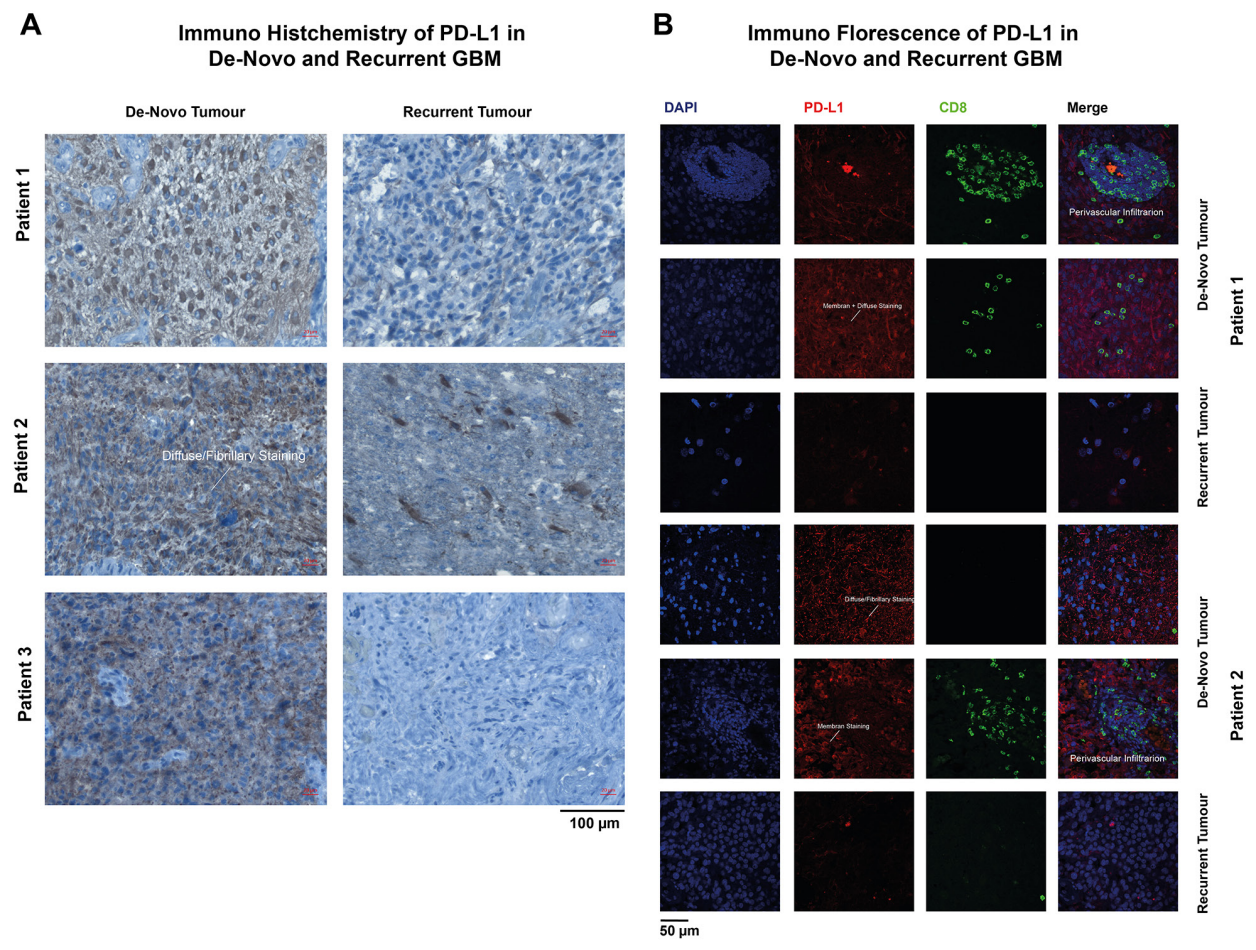
Immunohistochemistry (IHC) **(A)** and immunofluorescence (IF) **(B)** of de-novo and recurrent GBM. The arrows marked different types of PD-L1 expression patter.

In line with reported results [15], IHC and IF showed different types of PD-L1 expression. The majority of patients showed a diffuse/fibrillary staining type (75%), followed by the cytoplasmic (20%) and membranous type (5%) (Examples are given in Figure 3A-3B). Recurrent GBM showed a strongly reduced expression of PD-L1 (de-novo GBM 20.8% PD-L1 positive cells, recurrent 7.6% PD-L1 positive cells) and also less lymphocytic infiltration (de-novo GBM 17.5% CD8 positive cells, recurrent 5.3% CD8 positive cells), Figure 3A-3B.

### Regression analysis of PD-L1 expression and clinical features

**Figure 4:**
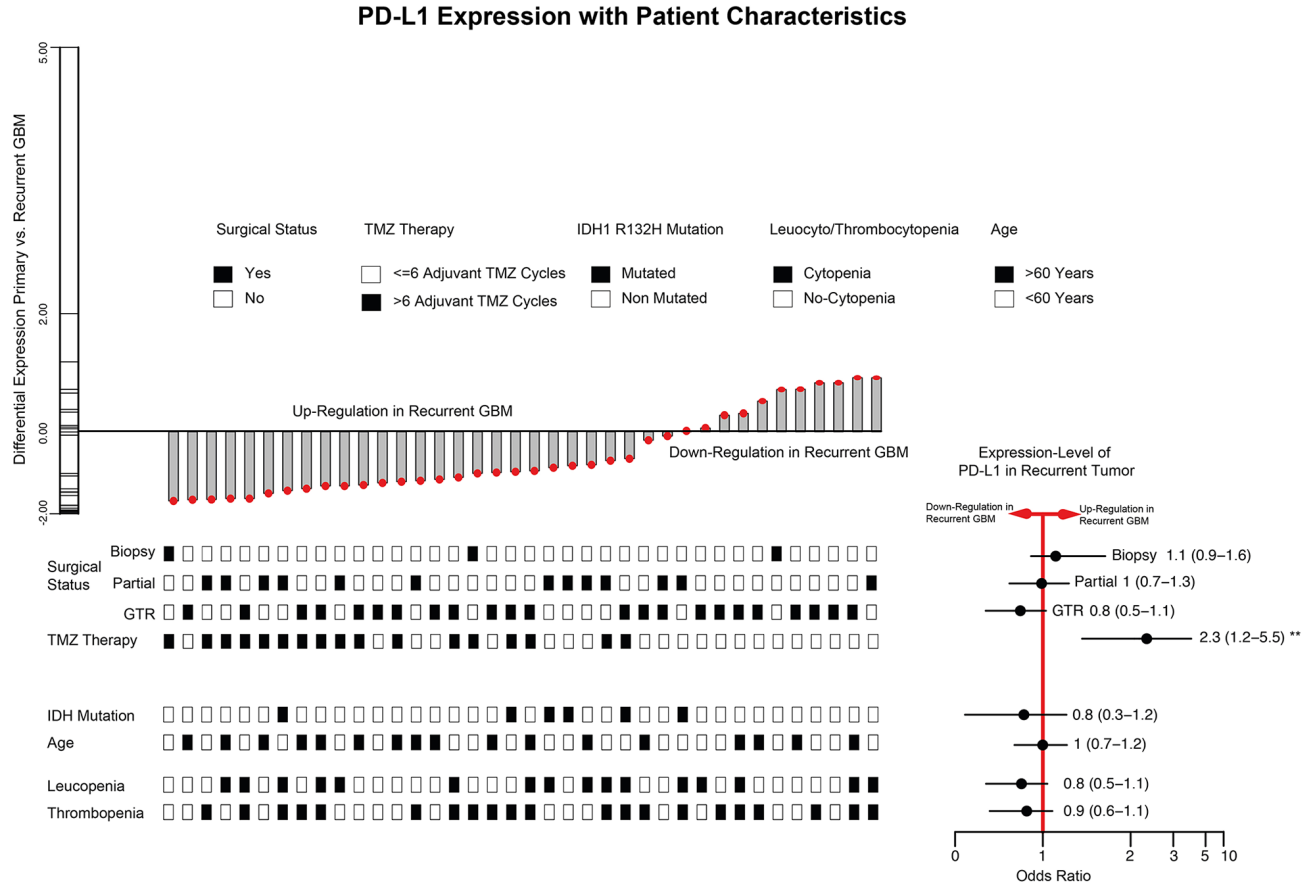
Barplot of PD-L1 expression differences between de-novo and 1^st^ recurrence (38 paired GBM patients) Clinical traits were visualized at the bottom of the barplot. The right bottom panel illustrated odds ratio and confidence interval. * (unvariete regression model p<0.05) ** (multivariate regression model p<0.05).

We identified 38 patients with matched de-novo and recurrent GBM and analysed associated clinical features, which promote the recurrent down-regulation of PD-L1 (Figure 4). First, a gross total resection (GTR) was realized in 23/38 patients, partial resection in 13/30 patient and only 3 patients received a biopsy in the de-novo stage. In a binominal generalized linear model, no significant connection between surgical procedure and *PD-L1* expression-level in the recurrent stage was predicted. Additionally, the age of the patients in the de-novo stage (Odds ratio 1 CI (0.7-1.2) p>0.05), *IDH1/2* mutation status (Odds ratio 0.8 CI (0.3-1.2) p>0.05) was not associated with a lower *PD-L1* expression in the recurrent tumour. Leuco- and thrombocytopenia regularly occurred in patients who underwent chemotherapy. However, our model could not predict any association with the expression-level of *PD-L1* and cytopenia complications. Finally, we analysed patients who received an extended temozolomide therapy (> 6 cycles, 17 patients) beyond the classical STUPP-protocol (6 cycles). Interestingly, a significant down-regulation of PD-L1 in the recurrent tumour was found in those patients (Odds ratio 2.3 CI (1.2-5.5) p=0.02), Figure 4.

## DISCUSSION

The purpose of this study was to analyse the occurrence of PD-L1 expression in recurrent glioblastoma. Being a “key-player “ of the immune escape mechanism of glioblastoma multiforme, PD-L1 is one of the most promising targets for future immune therapy [18]. In de-novo GBMs, PD-L1 is expressed in the majority of all tumours with a large variance of quantification within different studies based on IHC evaluations. We found 20.83% PD-L1 positive stained tumour cells with the Cell Signalling antibody (E1LRN) in combination with the SignalStain-Kit (Cell Signaling). Berghoff et al., reported much less PD-L1 positive cells (∼2%), which might be associated with different staining techniques and a different antibody [15]. The early results of the Checkmate 143 study reported nearly 80% PD-L1 positive stained cells, which reflect the wide range of PD-L1 IHC quantification [19]. In our study, the occurrence of PD-L1 enrichment was heterogeneously distributed within different regions of the tumor, which has already been described in the literature [14]. Additionally, the stainings showed several variations of PD-L1 staining patterns as shown in Figure 3. In fact, PD-L1 staining analysis is highly biased by the analyzed tumor region and observer. Especially the heterogeneity of PD-L1 enrichment within one biopsy is of importance, as it can lead to a false classification of patients depending on the examined area of the biopsy. This false classification might then even lead to therapeutic consequences in the future and decide on whether a patient is administered immune checkpoint inhibitors or not. To receive a more accurate impression of gene expression within the whole biopsy, mRNA expression levels seemed more robust. However, it must be taken into consideration that mRNA does not automatically result in protein but undergoes many posttranscriptional steps. A recent published analysis of PD-L1 regulation showed a high accuracy of PD-wL1 expression as a marker of immune driven molecular changes in malignant glioma [9]. For this reason, additional qRT-PCR was performed to analyze the PD-L1 expression level.

Up to today, little is known about recurrent GBM and the abundance of tumour-specific alterations has so far only partially been investigated. Since recurrent GBM are difficult to treat and show a high therapeutic resistance, there is variation in treatment strategies. Most of the recurrent tumours received several therapies (TMZ and radiation) and contained an increased number of mutations. Due to the increase of potential neoantigens, it is assumed that recurrent tumours should be more accessible for immune system recognition and attack. In contrast to the hypothesis that PD-L1 expression would increase as a reaction of the tumour to protect itself form a stronger immune response (immune-escape mechanism) in recurrent tumours, we found the opposite effect. PD-L1 mRNA expression and protein level were significantly reduced in recurrent tumours, which was confirmed by an additional analysis of 6 independent cohorts. Berghoff et al., (n=18 patients) reported a reduction of PD-L1 in 22%, a stable protein-level in 66.7% and only in 5.5% an increased PD-L1 within the recurrent tumour. PD-L1 expression was measured using IHC only, and staining was very heterogeneous within one sample especially in recurrent tumours. In line with the findings by Berghoff et al., the results from an analysis of CD8 and CD3 infiltration in de-novo and recurrent tumours showed reduced levels in recurrent tumours. However, Miyazaki and colleagues did not confirm the reduction of PD-L1 in a recurrent GBM in a small (n=16) cohort. Additionally, the cohort partially achieved immunotherapy, which biased the findings and hinder a clear interpretation of the results [16].

Finally, a regression model of PD-L1 expression and clinical data showed one significant parameter, which was associated with a down-regulation of PD-L1. The extended TMZ therapy (>6 cycles) might have a negative influence on the immune system activity, which then results in less immune infiltration within the tumor and consecutively leads to a decreased need for PD-L1 expression as immune-escape mechanism within the tumour. Other parameters for immune system malfunction like leuco- and thrombocytopenia (also connected to TMZ therapy) did not showed a significant connection to PD-L1 expression. The immune modulation function of TMZ in the context of immunotherapy was observed in different studies but is not well understood and needs to be explored in the future [20].

### Limitations

This study was limited by a relatively small number of de-novo and recurrent pairs, but achieved a number of 38 cases. With regard to the limited number of patients, a statistical power of 0.8 was achieved for all tests, which reflects the robustness of statistical testing. In conclusion, this study reported a reduction of PD-L1 expression in recurrent GBM, which might be caused by the immune modulating effect of TMZ therapy. The effect of PD-L1/PD-1 immune therapy of recurrent tumours is still unclear and needs accurate evaluation. As known from other cancer entities, PD-L1 occurrence and the therapeutic effect of PD-L1/PD-1 therapy are not necessarily connected. The exact mechanisms of PD-L1 regulation in de-novo and especially recurrent tumours will need more examination in future studies.

## MATERIALS AND METHODS

### Transcriptional data analysis of various datasets

Publicly available Level 3 TCGA (https://cancergenome.nih.gov/) data were used for analysis. Data were downloaded at the UCSC Cancer Genome Browser. Expression analysis was performed based on Agilent array data (TCGA GBM G4502A) for high-grade glioma. Additional datasets of recurrent GBM were downloaded from publicly available platforms (http://gliovis.bioinfo.cnio.es) [17].

### Tissue collection and histology

Tumor tissue was sampled from contrast enhancing regions identified by intraoperative neuronavigation (Cranial Map Neuronavigation Cart 2, Stryker, Freiburg, Germany) during tumor resection. The tissue was snap-frozen in liquid nitrogen immediately after resection and processed for further analysis. The local ethics committee of the University of Freiburg approved data evaluation and experimental design (protocol 100020/09 and 5565/15). The methods were carried out in accordance with the approved guidelines. Written informed consent was obtained from all patients. Descriptive statistics of the patient cohort was given in Table 1.

**Table 1:** Descriptive statistics of all patients in the analysis

	Patients (n, (%))
**Sex (%)**	
Female	21 (32.8)
Male	41 (67.2)
**Age (SD)**	52.92 (17.16)
**Radiotherapy (%)**	50 (78.1)
> 55Gy	46 (71.87)
< 55Gy	4
**Chemotherapy (%)**	64 (100)
TMZ	58 (90.6)
Lomustin	5 (7.8)
Tacrolimus	1 (1.56)
**Surgery (%)**	64 (100)
Gross-Total Resection	43 (67.1)
Partial Resection	14 (21.8)
Biopsy	7 (10.9)
**MGMT (%)**	60 (93.7)
Not Methylated	35 (54.68)
Methylated	25 (39.06)
Not Evaluated	8 (12.5)
**IDH 1/2 Mutation (%)**	64 (100)
Mutation	5 (7.8)
Wildtype	59 (92.8)
**Leukopenia (%)**	33 (51.5)
> Grade 3	3 (4.69)
**Thrombopenia (%)**	36 (56.3)
> Grade 3	2 (3.13)
**Long-Term Steroids**	9 (14.06)
**Immune Suppressive Therapy**	2 (3.13)

SD: Standard deviation

### Immunohistochemistry (IHC)

Tissue samples were fixed using 4% phosphate buffered formaldehyde and paraffin-embedded according to standard procedures. H&E staining was performed on 4 μm paraffin sections using standard protocols. Immunohistochemistry was applied using an autostainer (Dako) after heat-induced epitope retrieval in citrate buffer. IDH1 mutation was assessed by immunohistochemistry using an anti-IDH1-R132H antibody (1:20, Dianova). Immunohistochemistry was performed on 3 μm paraffin-embedded tissue sections after deparaffinization and heat-induced epitope retrieval in citrate buffer by using the SignalStain Kit by Cell Signaling according to the manufacturer’s instructions. As primary antibody, Anti-PD-L1-antibody (E1LRN by Cell Signaling) was applied to the tissue in a concentration of 1:200 and incubated overnight at 4°C. The next day, after application of SignalStain® Boost Solution and Secondary Antibody Solution, counterstaining with Meyer’s haemalaun solution was performed. The samples were then mounted and analyzed with an Olympus microscope. PD-L1 positive cells were counted in 6 high-fields (40x magnification) per slide and compared to the total number of cells in each field. From this data, the mean percentage of PD-L1 positive cells was calculated.

### Immunofluorescence (IF)

The following antibodies were used for Imm-munofluorescence: PD-L1 (E1LRN, Cell signaling 1:200) and CD8 (CD8/144B, ThermoFisher, 1:200). Primary antibodies were used at the concentration indicated by the manufacturers. Anti-Mouse and anti-Rabbit Alexa488- or Alexa594-conjugated (Life Technologies) were used as secondary antibodies. Alexa594/Alexa488-conjugated antibodies were used at 1:100 dilution. Pictures were acquired using a fluorescence microscope (FL10i, Olympus). Image quantification was performed by ImageJ and analyzed by R-software.

### Quantitative real time PCR

RNA was prepared using the All Prep DNA/RNA Protein Mini Kit (Qiagen) and used for first strand cDNA synthesis using random primers and SuperscriptIII Reverse Transcriptase (Invitrogen). RNA and cDNA concentration was controlled using the NanoDrop(Thermo Fisher) spectrophotometre. Quantitative real-time PCR (qRT-PCR) was performed using a SYBR Green PCR Master Kit (Applied Biosystems). Primersequence were: *PD-L1* (for (TGGCATTTGCTGAACGCATTT), rev (TGCAGCCAGGTCTAATTGTTTT)) and *S18* (for (TTTGCGAGTACTCAACACCA), rev (CC-ACACCCC-TTAATGGC))

### Statistical analysis

The primary endpoint was to determined the difference of mRNA expression between de-novo GBM and recurrence. Distribution and variances of all data was tested by Shapiro-Wilk test (p>0.05) to confirm normality. We tested the difference between de-novo and recurrent GBM by Wilcoxon signed-rank test (unpaired) and determined a 5% alpha-level. For the paired (de-novo/1^st^ recurrence) samples we used the paired Wilcoxon signed-rank test (IHC and mRNA expression). The validation cohort was normally distributed (Shapiro-Wilk p=0.3) and differences were tested by unpaired student’s t-test with an alpha-level of 5%. A binominal generalized linear model was used to find associated clinical parameters to up/down regulation of PD-L1 in the recurrence stage. Significance was calculated by chi-square with an alpha-level of 5%. All statistical analysis was performed with R-software.

## References

[1] Ostrom QT, Gittleman H, Liao P, Rouse C, Chen Y, Dowling J, Wolinsky Y, Kruchko C, Barnholtz-Sloan J (2014). CBTRUS Statistical Report: Primary Brain and Central Nervous System Tumors Diagnosed in the United States in 2007-2011. Neuro Oncol.

[2] Gilbert MR, Dignam JJ, Armstrong TS, Wefel JS, Blumenthal DT, Vogelbaum MA, Colman H, Chakravarti A, Pugh S, Won M, Jeraj R, Brown PD, Jaeckle KA (2014). A randomized trial of bevacizumab for newly diagnosed glioblastoma. N Engl J Med.

[3] Chinot OL, Wick W, Mason W, Henriksson R, Saran F, Nishikawa R, Carpentier AF, Hoang-Xuan K, Kavan P, Cernea D, Brandes AA, Hilton M, Abrey L, Cloughesy T (2014). Bevacizumab plus radiotherapy-temozolomide for newly diagnosed glioblastoma. N Engl J Med.

[4] Stupp R, Mason WP, van den Bent MJ, Weller M, Fisher B, Taphoorn MJ, Belanger K, Brandes AA, Marosi C, Bogdahn U, Curschmann J, Janzer RC, Ludwin SK (2005). Radiotherapy plus concomitant and adjuvant temozolomide for glioblastoma. N Engl J Med.

[5] Polivka J, Polivka J, Holubec L, Kubikova T, Priban V, Hes O, Pivovarcikova K, Treskova I (2017). Advances in Experimental Targeted Therapy and Immunotherapy for Patients with Glioblastoma Multiforme. Anticancer Res.

[6] Gatalica Z, Snyder C, Maney T, Ghazalpour A, Holterman DA, Xiao N, Overberg P, Rose I, Basu GD, Vranic S, Lynch HT, Von Hoff DD, Hamid O (2014). Programmed cell death 1 (PD-1) and its ligand (PD-L1) in common cancers and their correlation with molecular cancer type. Cancer Epidemiol Biomarkers Prev.

[7] Snyder A, Makarov V, Merghoub T, Yuan J, Zaretsky JM, Desrichard A, Walsh LA, Postow MA, Wong P, Ho TS, Hollmann TJ, Bruggeman C, Kannan K (2014). Genetic Basis for Clinical Response to CTLA-4 Blockade in Melanoma. N Engl J Med.

[8] Ohaegbulam KC, Assal A, Lazar-Molnar E, Yao Y, Zang X (2015). Human cancer immunotherapy with antibodies to the PD-1 and PD-L1 pathway. Trends Mol Med.

[9] Henrik Heiland D, Haaker G, Delev D, Mercas B, Masalha W, Heynckes S, Gabelein A, Pfeifer D, Stella Carro M, Weyerbrock A, Prinz M, Schnell O (2017). Comprehensive analysis of PD-L1 expression in glioblastoma multiforme. Oncotarget.

[10] He J, Hu Y, Hu M, Li B (2015). Development of PD-1/PD-L1 Pathway in Tumor Immune Microenvironment and Treatment for Non-Small Cell Lung Cancer. Sci Rep.

[11] Nickel GC, Barnholtz-Sloan J, Gould MP, McMahon S, Cohen A, Adams MD, Guda K, Cohen M, Sloan AE, LaFramboise T (2012). Characterizing mutational heterogeneity in a glioblastoma patient with double recurrence. PLoS One.

[12] Bouffet E, Larouche V, Campbell BB, Merico D, De Borja R, Aronson M, Durn C, Krueger J, Cabric V, Ramaswamy V, Zhukova N, Mason G, Farah R (2016). Immune checkpoint inhibition for hypermutant glioblastoma multiforme resulting from germline biallelic mismatch repair deficiency. J Clin Oncol.

[13] Champiat S, Ferté C, Lebel-Binay S, Eggermont A, Soria JC (2014). Exomics and immunogenics: Bridging mutational load and immune checkpoints efficacy. Oncoimmunology.

[14] Nduom EK, Wei J, Yaghi NK, Huang N, Kong LY, Gabrusiewicz K, Ling X, Zhou S, Ivan C, Chen JQ, Burks JK, Fuller GN, Calin GA (2016). PD-L1 expression and prognostic impact in glioblastoma. Neuro Oncol.

[15] Berghoff AS, Kiesel B, Widhalm G, Rajky O, Ricken G, Wöhrer A, Dieckmann K, Filipits M, Brandstetter A, Weller M, Kurscheid S, Hegi ME, Zielinski CC (2014). Programmed death ligand 1 expression and tumor-infiltrating lymphocytes in glioblastoma. Neuro Oncol.

[16] Miyazaki T, Ishikawa E, Matsuda M, Akutsu H, Osuka S, Sakamoto N, Takano S, Yamamoto T, Tsuboi K, Matsumura A (2017). Assessment of PD-1 positive cells on initial and secondary resected tumor specimens of newly diagnosed glioblastoma and its implications on patient outcome. J Neurooncol.

[17] Bowman RL, Wang Q, Carro A, Verhaak RGW, Squatrito M (2017). GlioVis data portal for visualization and analysis of brain tumor expression datasets. Neuro Oncol.

[18] McDermott DF, Atkins MB (2013). PD-1 as a potential target in cancer therapy. Cancer Med.

[19] Reardon DA, Sampson JH, Sahebjam S, Lim M, Baehring JM, Vlahovic G, Cloughesy TF, Strauss LC, Latek RR, Palatal P, Harrison CT, Voloshin AD, Padula Omuro AM (2016). Safety and activity of nivolumab (nivo) monotherapy and nivo in combination with ipilimumab (ipi) in recurrent glioblastoma (GBM): Updated results from checkmate-143. J Clin Oncol.

[20] Sengupta S, Marrinan J, Frishman C, Sampath P (2012). Impact of temozolomide on immune response during malignant glioma chemotherapy. Clin Dev Immunol.

